# Youth Football Players’ Psychological Well-Being: The Key Role of Relationships

**DOI:** 10.3389/fpsyg.2020.567776

**Published:** 2020-11-10

**Authors:** Eleonora Reverberi, Chiara D’Angelo, Martin A. Littlewood, Caterina Francesca Gozzoli

**Affiliations:** ^1^Department of Psychology, Catholic University of the Sacred Heart, Milan, Italy; ^2^School of Sport and Exercise Sciences, Liverpool John Moores University, Liverpool, United Kingdom

**Keywords:** psychological well-being, youth athletes, football, relationships, psychosocial approach

## Abstract

Well-being in youth sport is a growing topic in literature. Practicing sports at a youth level is recognized as an important opportunity for growth and development but also an experience that conversely can prove to be tiring and cause discomfort. Sometimes expectations and pressures make it a risky experience. This is emphasized even more when looking at very popular and spectacular sports, such as football in some European Countries; practicing football often solicits the hope of becoming champions one day and thus being able living thanks to the beloved sport. How do young Italian football practitioners feel? What role do relationships with significant others belonging to the world of sport and extra-sport play on the well-being of young athletes? On which specific aspects of psychological well-being (PWB) are these relationships based? Are there any differences between elite and amateurs levels? These are the questions upon which this paper focuses, considering a sample of young Italian football practitioners. Analysis reveals a strong and positive influence of some dimensions of the relationships with significant others on PWB, specifically team effort, coach closeness, and parental learning climate. Moreover, elite players perceive significantly better relationships than sub-elite and amateurs and have significantly higher levels of PWB. Those results provide a first evidence for the importance of good relationships within and outside sport for an effective development of youth football players since they positively influence players’ PWB, which is higher in elite players. It emerges the necessity to further investigate different aspects of PWB and to deepen the knowledge about the meaning of relationship in developmental athletes according to a psychosocial approach.

## Introduction

Practicing a sport, especially at a young age, can involve meetings and formative experiences or vice versa turn out to be a tiring experience and can sometimes also a cause of deep discomfort if not managed well (Sanchez-Martin, 2003; Holt et al., 2008; Balague et al., 2013)—relationships of trust versus feelings of loneliness, aggregation versus exclusion, a sense of improvement versus de-motivation and a sense of self-doubt. Furthermore, the more the level of competition increases, the more sports commitments increase and the conciliation with studies becomes more complicated. Usually the expectations of the young person and of the context increase.

The increasing demands from the sport setting during the developmental years of athletes challenge them with always increasing pressures, like more and more hours of training and competitions (Baker, 2003; Fraser-Thomas et al., 2008), along with the necessity to leave the family home at a very young age and to lead an “adult life” during a period of life which is particularly delicate for both their growth (physical, social, and psychological) and their athletic development (Balish and Côté, 2014). All these transitions (Wylleman and Lavallee, 2004, D’Angelo et al., 2017) can be hard to face without adequate relational support; such pressures could negatively impact on the development of athletes, in particular on their well-being.

For this reason, deepening the theme maintaining a focus on the well-being of young athletes seems relevant in coherence with a holistic perspective, and specifically with the psychosocial approach, which emphasizes how the well-being or malaise of each person is influenced by his life context and in particular by the quality of relationships with people significant to him/her.

How do young Italian football practitioners feel? What role do relationships with significant others belonging to the world of sport and extra-sport play on the well-being of young athletes? On which specific aspects of psychological well-being (PWB) are these relationships based? Are there any differences between elite and amateurs levels?

Using a psychosocial approach (Manzi and Gozzoli, 2009; Larsen et al., 2012; Gledhill et al., 2017), the present work examines the impact of the relationships youth football athletes have with significant others (within and outside sport) on their PWB, considering well-being as a basic condition moreover for good performance in sport (Rees, 2007; Larson et al., 2019). According to our perspective (Tseng and Seidman, 2007; Manzi and Gozzoli, 2009; Gozzoli et al., 2014; D’Angelo et al., 2018; Gozzoli et al., 2018), relationship is considered both a mutual bond among people—that can be a constraint and a resource for them—and also a set of specific meanings, values, and expectations assigned to it. Thus, considering sport practice under a psychosocial point of view means keeping in mind that young athletes are continuously involved in relationships that are create constrain among people that they give a meaning to.

Before presenting the empirical study, the state of the art of literature on the subject will be briefly presented.

Well-being is a complex multidimensional concept that has been studied mainly from two perspectives, namely, hedonic and eudaimonic. These can be considered two different parts of the same general concept of well-being, but their origins are different (Sirigatti et al., 2009; Huta and Ryan, 2010). Hedonic perspective defines well-being from a subjective point of view [subjective well-being (SWB); Diener, 2009], considering the cognitive and affective evaluation that people give about their lives as fundamental for their well-being (Diener et al., 2002). Eudaimonic perspective, instead, introduces the concept of PWB (Ryff and Keyes, 1995), which refers more to the possibility to reach human potential and to have the resources necessary to reach an optimal level of functioning in the long term (Ryff, 1989). Carole Ryff is the main reference author of eudaimonic PWB: she affirms that well-being is based more on the psychological abilities that people need to grow up and develop, that help them in facing effectively with life challenges and crisis (Ryff and Keyes, 1995). The author created the *multidimensional model of PWB*, which includes six dimensions (Ryff, 1989, 2013; Ryff and Keyes, 1995): *self-acceptance*, a positive attitude toward the oneself and one’s past life and experiences; *positive relations with others*, that is the ability to have an open and satisfying relationship with others; *autonomy*, or a sense of independence and self-determination in own’s life; *environmental mastery*, the competence to manage the daily activities; *purpose in life*, or the belief of a unique meaning to one’s life; *personal growth*, a positive attitude toward new experiences and the openness of mind.

Current research on well-being in sport has been carried out mainly by Carolina Lundqvist. Lundqvist suggested a model of well-being in èlite sport which is based on the union of subjective and PWB on both general and sport aspects of development (Lundqvist, 2011). The model lies on the awareness that athletes have two main areas of development—a “non-sporting/personal” area and a “sporting” one—and thus they could experience a different kind of well-being in each.

Lundqvist has deepened its studies especially in the field of elite sport (Lundqvist and Sandin, 2014) and it is to them that we have referred for the operationalization of the construct of well-being in our empirical study. The choice to apply the study to a population of young athletes led us to choose to investigate in particular PWB, the most interesting dimension from the point of view of its development in adolescence. In fact, previous studies have shown that PWB is associated with a more positive individual development, both as athletes and as person, a long-term involvement in sport, intrinsic motivation, and better coping strategies (Adie et al., 2012; Ivarsson et al., 2015; Cheval et al., 2017), which are fundamental conditions for developing high-level performance.

Ryff’s multidimensional model referred to the sports context as from the work of Lundqvist and Sandin (2014) defines the six dimensions of PWB as follows. Self-acceptance in sport is the self-awareness of personal strengths and weaknesses with a realistic evaluation of current performance level and future achievements, and the acceptance of the difference between the person and the athlete’s results. Positive relations with others both within and outside sport are considered a fundamental pillar of their serenity, and negative events in one of them often impact on the other one. Autonomy in sport means knowing how to regulate everyday behaviors and decisions without the help of others or their direct request, as well as the awareness of the responsibilities of being an athlete. Environmental control is seen as the ability to identify and use environmental resources to face everyday challenges (e.g., combine school and training) or unexpected ones (e.g., injuries). Meaning in (athletic) life implies the effort to be devoted to a specific and higher life goal through sport. Finally, personal growth involves the possibility to develop holistically, trying to connect all life’s areas and feeling that each area brings positive effects to the rest.

Studies on PWB in developing athletes are increasing only recently. For instance, Rongen et al. (2020) in a recently published work studied the psychological impact of living in academies of young footballers. These contexts, in fact, require a growing commitment to training and competitions (Elbe et al., 2005), constant monitoring of performance, highly structured full-time schedules, and numerous sacrifices compared to non-sporting life (school attendance, time with friends) (Christensen and Sorensen, 2009). All these elements are potentially stressful for a young adolescent (DiFiori et al., 2014; Bergeron et al., 2015; Sabato et al., 2016) and can also compromise athletic performance and PWB.

Van Rens et al. (2019) investigated the issue of well-being of young athletes in relation to their dual career paths, assuming the development of an academic identity as a protective factor with respect to the development of the well-being of young Australian athletes.

Studies such as those just mentioned therefore confirm a growing interest in the psychological literature on youth sport regarding PWB as a fundamental condition for the promotion of development and athletic performance.

The importance of relationships in the developmental path of young athletes is currently an increasingly important issue (Larson et al., 2019). In recent years, the role of social environment and relationships in the development of young athletes has become one of the most investigated topics in the field of sport psychology (Sheridan et al., 2014). Better relationships are linked to an easier recovery from injuries, positive sport participation, increased self-confidence, and better performance outcomes, and as a consequence, lower levels of burn-out (Rees, 2007; Sheridan et al., 2014; Larson et al., 2019). Ivarsson et al. (2015) suggested that players who perceive their environment to be supportive and have a focus on long-term development are less likely to suffer from stress and experience greater well-being.

Positive relationships with significant others (e.g., coaches, teammates, parents, or siblings) have been identified as one of the most important resources for young athletes’ development since Bloom (1985) and Côté’s (1999) research. For example, increases in perceived autonomy support from the coach over two competitive seasons have been related to increases in youth elite football players’ well-being and decreases in their ill being (Adie et al., 2012). In a longitudinal study among youth elite swimmers, a task-oriented parent-initiated motivational climate was positively related to decreases in trait anxiety over the competitive season (O’Rourke et al., 2011). In addition, the coach- and peer-created motivational climate has been related to youth athletes’ moral attitudes and well-being, with positive associations shown with a task oriented climate and negative associations shown with a performance-oriented climate (Alvarez et al., 2012; Ntoumanis et al., 2012).

In summary, literature highlights that the well-being of athletes is a dimension studied especially among elite athletes (Lundqvist, 2011) and studies that consider the well-being of youth athletes are recently increasing (Kipp and Weiss, 2013; Lundqvist and Raglin, 2015; Van Rens et al., 2019; Rongen et al., 2020; Thomas et al., 2020), research on the importance of a good relationship with the coach, teammates, and parents is huge but tends to consider the impact of relationships mainly on the performance and considering them individually (Jowett et al., 2012; Ntoumanis et al., 2012; Jowett and Shanmugam, 2016; Gledhill et al., 2017). Most of these mentioned researches on PWB of athletes are qualitative studies, mostly with retrospective designs; moreover, these studies have investigated each relationship individually, but no studies have yet studied the contemporary impact of all these relationships on well-being. Completely absent are studies on well-being in youth sport in the Italian context.

The proposed study aims to make a contribution to the understanding of the well-being of young players within the Italian context with the particular focus not so much on the influence of a specific relationship (with the coach, with parents), but on the intertwining, the set of different relationships. In our study, we tested the influence of relationships with significant others on the PWB of youth football players, comparing players of different competitive level (i.e., professional, semi-professional, and amateur), to identify possible differences.

## Materials and Methods

### Participants

The sample is made up of 415 male young soccer players from two professional (League A and B, *N* = 127), two semi-professional (League C, *N* = 162) and four amateur (*N* = 128) Italian youth soccer academies, aged between 14 and 20 years (*M*_age_ = 16.2, *SD* = 1.51), mainly situated in the north of Italy (see Table 1 for mean age and range of each category). The clubs involved in the research were selected by convenience, using personal contacts of the first three authors of this work.

**TABLE 1:** Mean age and range of each category.

	**Additional data on sample subgroups**				
	***N***	***M*_age_**	***DS***	**Min.**	**Max.**
Professional	122	16.61	1.36	14	20
Semi-professional	151	16.20	1.54	14	19
Amateur	124	15.89	1.53	14	19

The majority of them were born in Italy (91%), while a minority were foreign (8.4%) or had dual nationality (2.4%). Most players lived with their parents (87.6%), a minority in a specific residential structure provided by the club (6.7%) or with one parent (4.3%).

### Measures

#### Socio-Demographic Information

Participants were asked to give details about their age, month of birth, nationality (also for their parents), siblings, parental educational level, and some details related to sport, like sports practiced in the family, other sports practiced in the past, sports practiced by siblings, and current injuries.

#### Psychological Well-Being

Psychological well-being was measured using Ryff’s PWB Scale (Ryff, 1989; Ryff and Keyes, 1995; Italian version by Sirigatti et al., 2009). Ryff’s PWB Scale is composed of 18 items, rated on a four-point Likert scale, ranging from (1) “completely disagree” to (4) “completely agree.” It measures six dimensions, namely: self-acceptance (e.g., “In general, I feel confident and positive about myself”), ω = 0.529; positive relations with others (e.g., “I often feel lonely because I have few close friends with whom to share my concerns”), ω = 0.588; autonomy (e.g., “It’s difficult for me to voice my opinions on controversial matters”), ω = 0.459; environmental mastery (e.g., “I am good at juggling my time so that I can fit everything in that needs to get done”), ω = 0.430; purpose in life (e.g., “I am an active person in carrying out the plans I set for myself”), ω = 0.476; personal growth (e.g., “I think it is important to have new experiences that challenge how you think about yourself and the world”), ω = 0.409.

#### Relationship With the Coach

The Coach–Athlete Relationship Questionnaire (Jowett and Ntoumanis, 2004) is used to measure the link between athlete and the coach. The Coach–Athlete Relationship Questionnaire consists of 11 items that measure three dimensions: commitment (e.g., “I am committed to my coach”), ω = 0.800; closeness (e.g., “I like my coach”), ω = 0.836; and complementarity (e.g., “When I am coached by my coach, I am responsive to his/her efforts”), ω = 0.782. Answers were scored on a five-point Likert scale, ranging from (1) “not at all” to (5) “very much.”

#### Relationship With Teammates

The 21 version of the Peer Motivational Climate in Youth Sport Questionnaire (Ntoumanis and Vazou, 2005) was used. The Peer Motivational Climate in Youth Sport Questionnaire measures five dimensions, namely: focus on improvement (e.g., “On this team, most athletes… help each other to improve”), ω = 0.760; relatedness support (e.g., “On this team, most athletes… care about everyone’s opinion”), ω = 0.749; effort (e.g., “On this team, most athletes… encourage their teammates to try their hardest”), ω = 0.755; intrateam competition (e.g., “On this team, most athletes…look pleased when they do better than their teammates”), ω = 0.664; intrateam conflict (e.g., “On this team, most athletes…make negative comments that put their teammates down”), ω = 0.748. Items were scored on a five-point Likert scale, ranging from (1) “not at all” to (5) “very much.”

#### Relationship With Parents

We used the Parent-Initiated Motivational Climate Questionnaire (White et al., 1992). It is made of 28 items divided for father (14) and mother (14), which measure three dimensions: learning/enjoyment climate (e.g., “I feel that my mother/father… encourages me to enjoy learning new skills”), ωF = 0.544, ωM = 0.646; worry conductive climate (e.g., “I feel that my mother/father… makes me worried about performing skills that I am not good at”), ωF = 0.584, ωM = 0.619; success without effort climate (e.g., “I feel that my mother/father… believe that it is important for me to win without trying hard”), ωF = 0.661, ωM = 0.737. For all 28 items, players answer twice to the introductory segment “I feel that my mother/father…,” and items were scored on a five-point Likert scale, ranging from (1) “not at all” to (5) “very much.”

### Procedure

After gaining the approval from the ethical commission of the university, professional, semi-professional, and amateur soccer clubs were contacted in different ways, but all thanks to the personal knowledge of the Italian authors of the present work. After the club’s formal acceptance, a presentation of the research (e.g., a brochure with the main aims of the research, technical information about the duration of the data collection, and the contacts of the researchers) was sent by email. Managers used it to present the research to parents and coaches and give contacts for the researchers in case of questions from the participants. After gaining informed consent from participants or their parents, a session of group data collection was organized for each team before or after one of the weekly trainings. Before each data collection session, the researcher explained to the players the main aims of the research and the main tasks required (e.g., “To investigate the experience players were living in their development as soccer players and to understand the involvement and role of significant others”). The total duration of each data collection session was between 30 and 45 min.

### Data Analysis

#### Preliminary Analysis

Using SPSS 20.0 (IBM Corp, 2011), we tested items’ scores for normality and calculated the means and standard deviations for each variable (see Table 2). After that, we ran a confirmatory factor analysis using Mplus 7.11 (Muthén and Muthén, 1998–2017) for each scale.

**TABLE 2:** Mean and standard deviations for each variable considered in the whole sample.

	**M**	***SD***
PWB_composite	9.57	0.94
PWB_autonomy	9.32	1.52
PWB_meaning	9.47	1.70
PWB_self-acceptance	9.50	1.31
PWB_positive relation	9.92	1.55
PARENT_worry	3.42	0.64
PARENT_learn	3.82	0.53
COACH_composite	3.74	0.74
COACH_closeness	4.03	0.78
COACH_complementarity	3.75	0.76
COACH_committment	3.46	0.86
TEAM_learning	3.49	0.74
TEAM_effort	3.79	0.67
TEAM_intracompet	3.30	0.69
TEAM_intraconflict	2.83	0.86

#### Structural Equation Model on the Impact of Relationships on Psychological Well-Being

**FIGURE 1:**
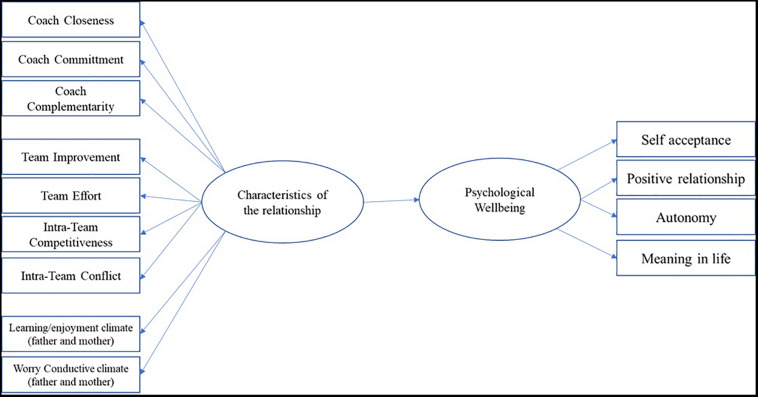
Hypothesized SEM on the impact of relationships on the psychological wellbeing of players.

Structural equation models (SEMs) are used in the analysis of behavioral data, as they make it possible to study the interrelationships between different latent factors and observed variables. They are particularly suitable for testing complex models in which the interactions provide for the inclusion of multiple variables and the presence of latent variables that cannot be measured directly. We adopted SEM because instead of studying singularly the impact of each relationship on PWB as in previous studies, we wanted to connect all the relationships with significant others and study their overall impact on the PWB of athletes. The SEM we tested using Mplus 7.11 (Muthén and Muthén, 1998–2017) is drawn in Figure 1: we form two latent variables (namely “Characteristics of the relationships” and “PWB,” reported in the circles) and their direct effects, which are composed of different observed variables (or the measurement model in the squares). The analysis of missing data patterns shows that the model considers only 302 subjects.

#### Analysis of Variance

To identify potential differences among the three competitive level groups (elite, subelite, and amateur) on the dimensions investigated (PWB, coach–athlete relationship, peer motivational climate in youth sport, parent-initiated motivational climate), we performed an ANOVA using SPSS 20.0.

## Results

### Preliminary Analysis

The PWB scale shows a non-normal distribution for some items: this could be imputed to the four-point Likert scale, which could lead to a positive–negative polarization of responses, found also in other works (Sirigatti et al., 2009); therefore, we treated factors as categorical when running the subsequent analysis. The CFA for the PWB Scale shows that only four factors over six emerged as reliable in our sample that are self-acceptance, positive relationships with others, autonomy, and purpose in life. Fit indices for this model were acceptable [χ^2^(48) = 100.635; *p* < 0.001; RMSEA = 0.051, *p* < 0.001; CFI = 0.924; WRMR = 0.912]. Factor loading of the remaining factors are between 0.30 and 0.77 and significant (*p* < 0.001).

The CFA for the Coach-Athlete Relationship Questionnaire shows a good internal consistency [χ^2^(41) = 136.473; *p* < 0.001; RMSEA = 0.075, *p* < 0.001; CFI = 0.940; SRMR = 0.043]. Factor correlation is between 0.93 and 0.97, and this could indicate collinearity problems between factors: the literature reveals a high correlation among those dimensions, and many studies try to validate a single factor structure without obtaining satisfactory results (Yang and Jowett, 2012, 2013). For the Peer Motivational Climate in Youth Sport-Questionnaire, the CFA shows that only four factors over five resulted in being reliable in our sample; therefore, in the analysis, we only considered focus on improvement, effort, intrateam competitiveness, intrateam conflict. Fit indices for this model were acceptable [χ^2^(122) = 220.649; *p* < 0.001; RMSEA = 0.044, *p* < 0.001; CFI = 0.941; SRMR = 0.069]. Factor loadings were between 0.35 and 0.75 and significative (*p* < 0.0001). Finally, for the Parent-Initiated Motivational Climate Questionnaire, the CFA reveals only two motivational climates emerged as reliable in our sample; therefore, we only considered a double factorial solution, composed of the learning/enjoyment climate and worry conductive climate (by father and by mother). Fit indices for this model were acceptable [χ^2^(154) = 282.513; *p* < 0.001; RMSEA = 0.045, *p* < 0.001; CFI = 0.934; SRMR = 0.062]. All results of the CFA are reported in Supplementary Tables 1–16.

Analyses of the mean and *SD* for each dimension considered in our analysis show that positive relationships with others has the highest score among the PWB dimensions (*M* = 9.92; *SD* = *1.55*), parental learning climate has the highest score among the two dimensions considered (*M* = 3.82; *SD* = 0.53), closeness with the coach has the highest score among the three dimensions of the scale (*M* = 4.03; *SD* = 78) and finally team effort has the highest score among all the other dimensions considered (*M* = 3.79; *SD* = 67). It is curious to note that all the variables with the highest mean score resulted also in impacting the SEM most, except for positive relationships with others. This could indicate that, overall, young players in the sample perceive having good relationships with others and that this doesn’t vary across competitive-level groups (see the ANOVA section).

### Structural Equation Model

#### Characteristics of Relationships and Psychological Well-Being

**FIGURE 2:**
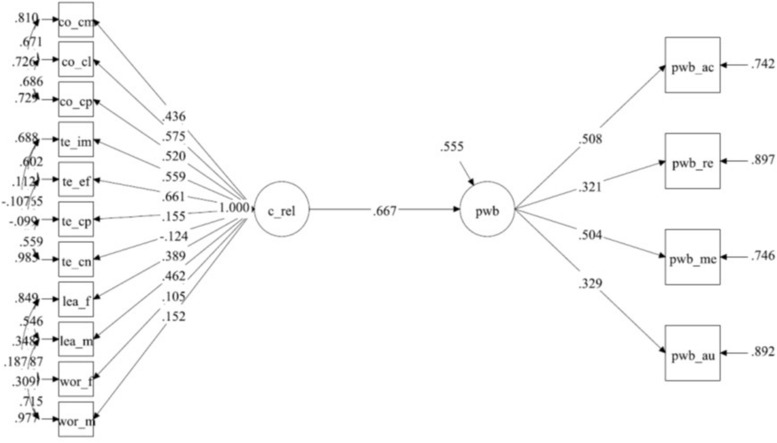
Resulted SEM on the impact of relationships on the psychological wellbeing of players.

The model fit information (reported in Table 3) together with the standardized model result of factor loading and factor correlations show an overall good fit for the model [χ^2^(74) = 142.917; *p* < 0.001; RMSEA = 0.047, *p* < 0.001; CFI = 0.963; SRMR = 0.049] (see Supplementary Table 17). The impact of the latent factor characteristics of relationships on the PWB is strong (0.667) and significative (see Figure 2 for the results of the analysis and Supplementary Table 17).

**TABLE 3:** Model fit information for the SEM.

**χ^2^ (*df*)**	***RMSEA***	***CFI***	***p***	***SRMR***
142.917 (74)	0.047	0.963	0.0001	0.049

Factor loading results (see Table 4) show that self-acceptance (0.508) and meaning in life (0.504) are the most significative dimensions in the PWB factor, while team effort (0.661), coach closeness (0.575), and team improvement (0.559) are the most important dimensions in the characteristics of relationships factor. The parental approach that mostly impact on the PWB factor was learning climate, promoted especially by mothers (0.462). Coach complementarity (0.520) and commitment (0.436) were only secondary dimensions in this model, while team conflict and worry conductive climate results were non-significant for father, while worry conductive climate has a minimal factor loading for mother (0.152, *p* < 0.05), as well as team competitiveness (0.155, *p* < 0.05).

**TABLE 4:** Estimated factor loading in the SEM.

**Dimensions of the latent factors**	**Estimated factor loading**
Self-acceptance	0.508***
Positive relationship	0.321***
Meaning in life	0.504***
Autonomy	0.329***
Coach commitment	0.436***
Coach closeness	0.575***
Coach complementarity	0.520***
Team improvement	0.559***
Team effort	0.661***
Intrateam competitiveness	0.155*
Intraream conflict	–0.124
Learning/enjoyment climate (father)	0.389***
Learning/enjoyment climate (mother)	0.462***
Worry conductive climate (father)	0.105
Worry conductive climate (mother)	0.152*

*p < 0.05.

***p < 0.001.

All in all, results confirm our hypothesis about the importance of relationships for the well-being of young athletes. In particular, there are some very specific features within the relationships with significant others that positively impact on athlete’s PWB, specifically on self-acceptance and meaning in life dimensions.

### Analysis of Variance Among Three Competitive Groups of Players

A comparison between the three competitive groups (elite, subelite, and amateur) was performed by an ANOVA (see Supplementary Tables 18–25). Next to each constituent dimension, we also calculated and compared groups on a composite score for the following dimensions: PWB, formed by a mean of the four significant constituent dimensions scores (α = 0.50); parent-initiated motivational climate, namely, respectively parental worry (α = 0.77) and parental learning motivational climate (α = 0.83) formed by the mean of the mother and father scores; at each dimension, the coach–athlete relationship (α = 0.90) formed by the mean of the three constituent dimensions.

ANOVA results were significant for the following dimensions: meaning in life, *F*(2,401) = 11.143, *p* < 0.001, autonomy, *F*(2,394) = 9.943, *p* < 0.001, and composite PWB, *F*(2,378) = 10.601, *p* < 0.001; parental worry motivational climate *F*(2,367) = 6.463, *p* < 0.002 and parental learning motivational climate *F*(2,367) = 6.174, *p* < 0.002, coach–athlete closeness *F*(2,408) = 6.041, *p* < 0.003, and finally ANOVA on peer motivational climate show a difference only in intrateam conflict, *F*(2,399) = 7.601, *p* < 0.001.

*Post hoc* test (HSD Tukey) reveals that composite PWB in elite (*M* = 9.85, *SD* = 0.85) is significantly higher than amateur (*M* = 9.31, *SD* = 0.94); autonomy in elite (*M* = 9.72, *SD* = 1.49) is significantly higher than amateur (*M* = 8.87, *SD* = 1.51); meaning in life in elite (*M* = 9.95, *SD* = 1.36) is significantly higher than amateur (*M* = 8.95, *SD* = 1.65). For the dimension intrateam conflict, results show that amateurs (*M* = 3.05, *SD* = 0.85) were significantly higher than elite (*M* = 2.63, *SD* = 0.70). For the coach–athlete relationship, we obtained a statistically significant difference only in the dimension of closeness, where amateurs (*M* = 3.84, *SD* = 0.95) were only lower than subelite (*M* = 4.14, *SD* = 0.64). Finally, regarding parent-initiated motivational climate, amateurs (*M* = 3.25, *SD* = 0.70) were significantly lower than elite (*M* = 3.55, *SD* = 0.58) on the worry motivational climate and also on the learning motivational climate, where amateur (*M* = 3.68, *SD* = 0.60) were lower than elite (*M* = 3.91, *SD* = 0.48) (see Supplementary Tables 18–25 for complete results of the ANOVA).

## Discussion

In this study, we wanted to examine how the relationships with significant others influenced the PWB of young soccer players. We analyzed for the first time the combined influence of three main significant others, namely, the coach, team, and parents. The literature shows that each of them is important for players’ well-being, but a comprehensive study of their combined influence has not been done until now.

Results of our analysis confirmed our hypothesis about the combined influence of relationships with significant others on the PWB of young athletes, in particular on their self-acceptance and their sense of having a purpose in life. Lundqvist (2011) described self-acceptance in athletes as their self-awareness of strengths and weaknesses, a realistic evaluation of current performance level and future achievements, and the acceptance of the difference between the person and the athlete’s results, while purpose in life was described as a sensation that implies the effort to be devoted to a specific and higher life goal through sport. Our analysis showed that perceiving effort and a focus on improvement within the team, having a close relationship with the coach and the promotion of a learning attitude by parents strongly influence players’ PWB, specifically to enhance their self-acceptance and sense of purpose in life. Moreover, our analysis also revealed that this influence was particularly strong in those players who were enrolled in professional and semi-professional clubs: this can be a possible sign of a very high degree of sensitivity toward the importance of relationships in those contexts.

Let us now explore the meaning that these specific characteristics of relationships can have in relation to the promotion of PWB in a young player. First, young players emphasize the importance of teammates’ motivational climate in their developmental path, more than the current research seems to have investigated (Holt et al., 2008; Fry and Gano-Overway, 2010; Bruner et al., 2014; García-Calvo et al., 2014; Sheridan et al., 2014). Our analysis showed that young players consider effort and focus on improvement within their team as the most effective relational elements for their PWB, being even more important than their coach or parents. A task-oriented motivational climate leads athletes to appreciate improvements, increase efforts, and consider errors as a part of the learning process and growth, leading everyone to be more satisfied with their sporting outcomes and remain engaged in sport for longer (Jõesaar et al., 2012; García-Calvo et al., 2014). This result seems to be particularly important in a team sport like football, as the improvement of one player could lead to the improvement of the overall team, thus supporting also the development of leadership and social skills (i.e., the ability to develop effective relationships with others). In other studies, Bruner et al. (2014) have shown how a higher level of task and social cohesion lead to more positive youth development, specifically in personal and social skills, initiative, goal setting, cognitive skills, and lower levels of negative experiences. Moreover, the ability to stay focalized on improvement and showing effort are also important when facing difficulties or important changes in life, like career transitions from junior to senior, specifically as a resource within sport context and as part of coping skills in relying on social support (Drew et al., 2019). Such findings support the importance of deepening the role of the peer motivational climate in the development of young athletes, especially in team sports (Ntoumanis et al., 2007, 2012).

Second, results highlighted the role of the coach–athlete relationship in promoting PWB. In particular, the relevance of the emotional closeness among other dimensions shows that feeling close to the coach can positively influence not only the performance (Jowett et al., 2012) but also the PWB of youths (Jowett and Poczwardowski, 2007; Davis et al., 2013; Jowett and Shanmugam, 2016). In general, the literature affirmed that athletes who perceive higher closeness, cooperation, and commitment with the coach also perceive their coaches to be more task-oriented (Olympiou et al., 2008). This could be more effective for their athletic career since such orientation allows sport engagement and continuation for a time, better and more effective goal setting, and higher levels of satisfaction from sport participation. Nevertheless, the quality of coach–athlete relationship has been found to be more effective within a long-term timeframe: thus, the longer the relationship, the better the results are (Jowett and Nezlek, 2012). In the clubs where data were collected—and in general in Italian football clubs—coaches usually change the team they train every year; thus both players and coaches need the ability to create a positive relationship within a very short time frame. If such an ability could be easier for adults, this would not be the same for adolescents who need to be supported in such aspects of development, especially in early adolescence (Wylleman et al., 2013).

Third, the parental motivational climate that promotes learning is considered as the most supportive for PWB. Specifically, elite players perceive their parents as more supportive for that climate than other groups do. In general, both parents emphasize a learning climate, as suggested by the results of previous studies. Those studies consider the motivational climate promoted by parents as a precursor of self-determined motivation toward sport, engagement, and higher levels of satisfaction with sport (White, 1998; White et al., 1998; Salselas and Marquez, 2009; Kolayiş et al., 2017). Moreover, parent-initiated motivational climate was found to be a significant predictor of late-season self-esteem, trait anxiety, and autonomous regulation, even higher than the coach-initiated motivational climate (O’Rourke et al., 2014). Interestingly, we found a difference in the weight of father and mother promotion of motivational climate, and this appears to be a curious emerging issue regarding the different parental approaches to sport (Wuerth et al., 2004; Kolayiş et al., 2017).

The results of our analysis allowed us to do another more general reflection. Adolescence is the period of life when new models are looked for to exit from the parental idealization phase and find new adults to trust and aspire to as role models. Coaches are the main landmark for youth in their sporting career: they are responsible for selecting players, organizing training to develop the best, deciding players for matches, and many other aspects that can help players to progress in their career, even more than parents. Our results show that coaches, next to teammates, assume a key role within the developmental path of young athletes as the relationship with them has been considered necessary for their PWB and sense of growth. Therefore, both coaches and parents need to be aware of such issues since they can be supported in developing a set of new relational skills to deal with athletes in this delicate phase-of-life transition.

Results of the comparison of the three competitive-level groups confirm that elite players have higher levels of PWB. It seem to confirm the recent study by Rongen et al. (2020), and we think it can be explained as follows: although the life of elite young footballers is busy and demanding, the value and achievement for them is high, they are in the place where they would like to be (generally top club academies), doing what they love more.

Specifically in our study elite athletes perceive higher levels of meaning in life, have higher levels of closeness with their coach, and also perceive that their parents promote a learning climate in sport more. Such results underline that not only relationships impact the PWB of players but also that elite ones have higher levels of PWB and better kinds of relationships with significant others. We consider this as evidence of our initial hypothesis—having better relationships within and outside sport can be considered as some of the psychosocial factors that support players to develop more effectively, as they promote the PWB, which in turn supports them in staying involved in sport and facing transitions and difficulties better.

### Some Final Reflections

According to a psychosocial approach, sport is a complex relational space (Sanchez-Martin, 2003; Holt et al., 2008) where relationships play a fundamental role in athletes’ development and throughout their entire athletic career. PWB, as formulated by Ryff and studied in sport by Lundqvist (Lundqvist, 2011; Lundqvist and Sandin, 2014) seems to be a useful basic condition for positive and effective development of young athletes.

The study is of course a first exploration of some constructs according to the need to consider the importance of PWB in the development of youth in sport, specifically for youth athletes involved on path of talent development (in our study, the elite group of football players). In the future, it could also be interesting to deepen the qualitative study of the meanings of well-being for this specific category of athletes.

Therefore, the study also shows some limits. The sample involved in the data collection is made of players from clubs situated mainly in the north of Italy: this could influence the representativeness of the sample with respect to the Italian population. Moreover, the literature has suggested some differences in males and females’ perceptions of the motivational climate induced by parents (Vesković et al., 2013; Gledhill and Harwood, 2014). It could be interesting to expand the sample and also involve female soccer players in order to compare the results with those of males. A third limitation is linked to the fact that we explored a developmental topic without using a longitudinal method. It would be of great interest to introduce a longitudinal methodology to investigate the process of talent development and eventual changes in relationships’ characteristics. Moreover, it would be of great interest to further explore the role of individual psychological characteristics, as for example motivational orientation on PWB.

## Data Availability Statement

The raw data supporting the conclusions of this article will be made available by the authors, without undue reservation.

## Ethics Statement

The studies involving human participants were reviewed and approved by the Commissione Etica per la Ricerca in Psicologia (CERPS) of the Catholic University of the Sacred Heart, Milan. Written informed consent to participate in this study was provided by the participants, and where necessary, the participants’ legal guardian/next of kin.

## Author Contributions

ER, CD’A, and CG conceived the study and the hypothesis. ER collected the data and run the analysis. CD’A and ER wrote the first draft of the manuscript, while CG and ML supervised the work. All authors contributed to the article and approved the submitted version.

## Conflict of Interest

The authors declare that the research was conducted in the absence of any commercial or financial relationships that could be construed as a potential conflict of interest.
