# Supplementary material for: Youth Football Players’ Psychological Well-Being: The Key Role of Relationships
Source: Front Psychol. 2020 Nov 10;11:567776. doi: 10.3389/fpsyg.2020.567776 (PMC7683523; doi:10.3389/fpsyg.2020.567776)
Supplement: Supplementary file 1 [file Data_Sheet_1.pdf]

Supplementary Material

Preliminary analysis

Ryff's Psychological Well-Being Scale (Ryff, 1989; Ryff & Keyes, 1995), RPWB-S

Factor	M *(SD)*	*McDonald ω*
**Self-acceptance**	9.49 *(1.31)*	.529
**Positive relationship with others**	9.92 *(1.55)*	.588
**Autonomy**	9.31 *(1.52)*	.459
**Purpose in Life**	9.46 *(1.70)*	.476
**Personal Growth**	9.99 *(1.34)*	.409
**Environmental mastery**	9.18 *(1.56)*	.430

Table 1 - Factor scores for RPWB-S and McDonald Omega

	N	M *(SD)*	Skewness		Kurtosis	
PSYWB1	415	3,10 *(.62)*	-,314	,120	,407	,239
PSYWB2	412	3,16 *(.66)*	-,239	,120	-,593	,240
PSYWB3	411	1,79 *(.70)*	,534	,120	-,117	,240
PSYWB4	411	3,27 *(.68)*	-,580	,120	-,053	,240
PSYWB5	412	1,43 *(.73)*	1,650	,120	1,960	,240
PSYWB6	410	3,40 *(.62)*	-,640	,121	,086	,240
PSYWB7	414	3,16 *(.56)*	-,073	,120	,245	,239
PSYWB8	410	1,93 *(.81)*	,780	,121	,341	,240
PSYWB9	412	1,75 *(.81)*	,936	,120	,346	,240
PSYWB10	415	3,04 *(.69)*	-,481	,120	,418	,239
PSYWB11	414	3,23 *(.64)*	-,472	,120	,283	,239
PSYWB12	412	2,82 *(.81)*	-,320	,120	-,341	,240
PSYWB13	413	3,08 *(.69)*	-,538	,120	,542	,240
PSYWB14	407	1,91 *(.74)*	,647	,121	,442	,241
PSYWB15	411	3,14 *(.91)*	-,867	,120	-,075	,240
PSYWB16	412	3,33 *(.67)*	-,800	,120	,699	,240
PSYWB17	416	3,31 *(.70)*	-,645	,120	-,262	,239
PSYWB18	415	1,85 *(.79)*	,688	,120	,013	,239
PSYWB5R	412	3,57 *(.73)*	-1,650	,120	1,960	,240
PSYWB8R	410	3,07 *(.81)*	-,780	,121	,341	,240
PSYWB9R	412	3,25 *(.81)*	-,936	,120	,346	,240
PSYWB14R	407	3,09 *(.74)*	-,647	,121	,442	,241
PSYWB18R	415	3,15 *(.79)*	-,688	,120	,013	,239
PSYWB3R	411	3,21 *(.70)*	-,534	,120	-,117	,240

Table 2 - Item distribution for RPWB-S

Model	χ^2 *(df)*	*RMSEA*	*CFI*	*WRMR*
**18-items, 6 factors**	262.285 *(120)*	.053	.880	1.069
**15- items, 5 factors**	186.296 *(80)*	.057	.901	1.038
**12-items, 4 factors**	100.635 *(48)*	.051	.924	.912

Table 3 - CFA results for the RPWB-S tested models

Factor correlations 12 items, 4 factors	1	2	3	4
**Self-acceptance**	1			
**Positive relationship with others**	.305***	1		
**Autonomy**	.256**	.201*	1	
**Purpose in Life**	.535***	.215**	.605***	1
p < .05, ** p < .01, p < .001**				

Table 4 - Factor correlation for RPWB-S final model 12 items, 4 factors solution

Coach – Athlete Relationship Questionnaire (Jowett, 2004), C-ARQ

Factor	M *(SD)*	*ω*
**Commitment**	3.46 *(.86)*	.800
**Closeness**	4.03 *(.78)*	.836
**Complementarity**	3.75 *(.76)*	.782

Table 5 - Factor scores for C-ARQ and McDonald Omega

	N	M *(SD)*	Skewness		Kurtosis	
ALL1	412	3,77 *(1.06)*	-,878	,120	,408	,240
ALL2	411	3,35 *(1.02)*	-,370	,120	-,205	,240
ALL3	412	3,99 *(.92)*	-1,064	,120	1,311	,240
ALL4	413	3,95 *(.96)*	-,858	,120	,535	,240
ALL5	410	3,62 *(1.02)*	-,467	,121	-,324	,240
ALL6	412	3,68 *(.99)*	-,544	,120	,048	,240
ALL7	413	4,45 *(.86)*	-1,766	,120	2,969	,240
ALL8	409	3,42 *(1.0)*	-,272	,121	-,162	,241
ALL9	413	3,79 *(.94)*	-,658	,120	,297	,240
ALL10	412	3,97 *(.97)*	-,895	,120	,441	,240
ALL11	410	3,56 *(1.1)*	-,605	,121	-,121	,240

Table 6 - Item distribution for C-ARQ

Factor	χ^2 *(df)*	*RMSEA*	*CFI*	*SRMR*
**Commitment** **Closeness** **Complementarity**	136.473 (41)	.075	.940	.043

Table 7 - CFA results for the C-ARQ

Factor correlations	1	2	3
**Commitment**	1		
**Closeness**	.931**	1	
**Complementarity**	.971**	.961**	1
p < .05, ** p < .01, p < .001**			

Table 8 - Factor correlations for C-ARQ

Peer Motivational Climate in Youth Sport Questionnaire (Ntoumanis & Vazou, 2005), PMCYS-Q

Factor	M *(SD)*	*McDonald ω*
**Improvement**	3.49 *(.74)*	.760
**Relatedness support**	3.59 *(.77)*	.749
**Effort**	3.79 *(.67)*	.755
**Intra team competition**	3.29 *(.69)*	.664
**Intra team conflict**	2.82 *(.86)*	.748

Table 9 - Factor scores for PMCYS-Q and McDonald Omega

	N	M *(SD)*	Skewness		Kurtosis	
PeMC1	410	3,57 *(1.0)*	-,429	,121	-,212	,240
PeMC2	408	3,55 *(.91)*	-,338	,121	,065	,241
PeMC3	409	3,75 *(.91)*	-,550	,121	-,011	,241
PeMC4	408	3,62 *(1.0)*	-,476	,121	-,445	,241
PeMC5	410	2,72 *(1.1)*	,440	,121	-,544	,240
PeMC6	408	3,45 *(.96)*	-,241	,121	-,397	,241
PeMC7	411	3,72 *(.96)*	-,483	,120	-,153	,240
PeMC8	410	4,06 *(.83)*	-,664	,121	,167	,240
PeMC9	410	3,46 *(.99)*	-,253	,121	-,416	,240
PeMC10	410	2,95 *(1.1)*	,129	,121	-,909	,240
PeMC11	410	3,66 *(1.0)*	-,432	,121	-,497	,240
PeMC12	410	3,48 *(.98)*	-,262	,121	-,402	,240
PeMC13	412	4,09 *(.86)*	-,756	,120	,179	,240
PeMC14	408	3,37 *(1.1)*	-,243	,121	-,690	,241
PeMC15	407	3,09 *(1.0)*	-,021	,121	-,593	,241
PeMC16	406	3,32 *(.96)*	-,136	,121	-,338	,242
PeMC17	403	3,62 *(.97)*	-,415	,122	-,287	,243
PeMC18	404	3,45 *(.95)*	-,323	,121	-,189	,242
PeMC19	406	2,53 *(1.2)*	,466	,121	-,742	,242
PeMC20	405	2,60 *(1.1)*	,387	,121	-,703	,242
PeMC21	405	3,46 *(1.0)*	-,344	,121	-,385	,242

Table 10 - Item distribution for PMCYS-Q

Model	χ^2 *(df)*	*RMSEA*	*CFI*	*SRMR*	*AIC*	*BIC*
**5 factors**	458.705 *(179)*	.062	873	.079	22047.946	22341.481
**4 factors**	357.618 *(129)*	.066	.863	.080	19322.813	19564.074
**4 factors + correlations among items 1-3; 16-1; 17-16; 20-19; 20-5; 16-6; 20-10**	220.649 *(122)*	.044	.941	.069	19162.055	19431.464
p < .05, ** p < .01, p < .001**						

Table 11 - CFA results for the PMCYS-Q

Factor correlations	1	2	3	4
**Improvement**	1			
**Effort**	.938***	1		
**Intra-team competitiveness**	.334***	.419***	1	
**Intra-team conflict**	-.800	-.068	.608***	1
p < .05, ** p < .01, p < .001**				

Table 12 - Factor correlations for PMCYS-Q

Parent-Initiated Motivational Climate (White, Duda & Hart, 1992), PIMC-Q

Factor	M *(SD)*	*ω*
**Learning/Enjoyment climate father**	3.81 *(.56)*	.544
**Worry Conductive climate father**	3.37 *(.67)*	.584
**Success without effort climate father**	2.34 *(.69)*	.661
**Learning/Enjoyment climate mother**	3.82 *(.60)*	.646
**Worry Conductive climate mother**	3.46 *(.71)*	.619
**Success without effort climate mother**	2.40 *(.81)*	.737

Table 13 - Factor scores for PIMC-Q and McDonald Omega

	N	M *(SD)*	Skewness		Kurtosis	
PIMC_P1	409	4,40 *(.79)*	-1,375	,121	1,964	,241
PIMC_P2	409	4,01 *(.97)*	-,857	,121	,281	,241
PIMC_P3	405	2,99 *(1.01)*	,043	,121	-,361	,242
PIMC_P4	405	4,12 *(.79)*	-,886	,121	,998	,242
PIMC_P5	407	3,71 *(1.05)*	-,700	,121	-,082	,241
PIMC_P6	403	2,79 *(.94)*	,101	,122	-,271	,243
PIMC_P7	407	3,79 *(.96)*	-,648	,121	,138	,241
PIMC_P8	405	3,58 *(1.2)*	-,474	,121	-,734	,242
PIMC_P9	403	1,76 *(.97)*	1,366	,122	1,523	,243
PIMC_P10	405	3,55 *(1.01)*	-,450	,121	-,232	,242
PIMC_P11	403	2,84 *(1.18)*	,179	,122	-,909	,243
PIMC_P12	397	3,16 *(1.02)*	-,103	,122	-,354	,244

PIMC_P13	405	2,72 *(1.06)*	,318	,121	-,563	,242
PIMC_P14	401	1,85 *(.98)*	1,225	,122	1,132	,243
PIMC_M1	401	4,47 *(.78)*	-1,797	,122	3,934	,243
PIMC_M2	399	4,12 *(1.00)*	-1,073	,122	,591	,244
PIMC_M3	395	2,95 *(1.08)*	,103	,123	-,580	,245
PIMC_M4	400	4,15 *(.84)*	-1,022	,122	1,187	,243
PIMC_M5	396	3,81 *(1.06)*	-,643	,123	-,390	,245
PIMC_M6	397	2,74 *(1.04)*	,213	,122	-,489	,244
PIMC_M7	397	3,62 *(1.00)*	-,600	,122	-,115	,244
PIMC_M8	398	3,62 *(1.12)*	-,606	,122	-,636	,244
PIMC_M9	395	1,99 *(1.11)*	1,082	,123	,421	,245
PIMC_M10	397	3,71 *(.94)*	-,478	,122	-,070	,244
PIMC_M11	392	2,92 *(1.2)*	,060	,123	-,923	,246
PIMC_M12	390	3,22 *(1.0)*	-,133	,124	-,406	,247
PIMC_M13	394	2,86 *(1.1)*	,057	,123	-,732	,245
PIMC_M14	393	1,98 *(1.08)*	1,128	,123	,649	,246

Table 14 - Item distribution for PIMC-Q

**Model**	χ^2 *(df)*	***RMSEA***	***CFI***	***SRMR***	***AIC***	***BIC***	***Sample BIC***
**3 factors each parent**	676.112 *(321)*	.052	.886	.082	28431.729	28887.467	28528.888
**2 factors each parent**	282.513 *(154)*	.045	.934	.062	20429.485	20736.000	20494.831

Table 15 - CFA results for the PIMC-Q

**Factor correlations**	**1**	**2**	**3**	**4**
**Learning/Enjoyment climate father**	1			
**Worry Conductive climate father**	.521***	1		
**Learning/Enjoyment climate mother**	.655***	.433***	1	
**Worry Conductive climate mother**	.329***	.729***	.536***	1
p < .05, ** p < .01, p < .001***				

Table 16 - Factor correlations for PIMC-Q

Structural Equation Model results

**Factor**	**M *(SD)***
**Coach Commitment**	3.45 *(.86)*
**Coach Closeness**	4.03 *(.78)*
**Coach Complementarity**	3.75 *(.76)*

**Team Improvement**	3.49 *(.74)*
**Team Effort**	3.79 *(.66)*
**Intra-Team Competitiveness**	3.30 *(.68)*
**Intra-Team Conflict**	2.82 *(.86)*
**Learning/enjoyment climate (father)**	3.80 *(.56)*
**Learning/enjoyment climate (mother)**	3.82 *(.60)*
**Worry Conductive climate (father)**	3.37 *(.67)*
**Worry Conductive climate (mother)**	3.46 *(.71)*
**Self-acceptance**	3.16 *(.43)*
**Positive relationship**	3.30 *(.51)*
**Autonomy**	3.10 *(.50)*
**Meaning in life**	3.15 *(.56)*

Table 17 – Factor scores

One-way Analysis of Variance on competitive levels of players

	**G1 Elite**		**G2 Sub-elite**		**G3 Amateurs**	
	**M**	***SD***	**M**	***SD***	**M**	***SD***
**PWB_composite**	9.85	*.85*	9.56	*.94*	9.31	*.94*
**PWB_autonomy**	9.72	*1.49*	9.37	*1.46*	8.87	*1.51*
**PWB_meaning**	9.95	*1.36*	9.50	*1.85*	8.95	*1.65*
**PWB_self-acceptance**	9.75	*1.25*	9.41	*1.32*	9.35	*1.31*
**PWB_positive relation**	9.93	*1.47*	9.93	*1.51*	9.91	*1.68*
**PARENT_worry**	3.55	*.58*	3.46	*.62*	3.25	*.70*
**PARENT_learn**	3.91	*.48*	3.87	*.49*	3.68	*.60*
**COACH_composite**	3.72	*.67*	3.85	*.64*	3.63	*.89*
**COACH_closeness**	4.10	*.64*	4.14	*.70*	3.84	*.95*
**COACH_complementarity**	3.67	*.69*	3.88	*.68*	3.67	*.90*
**COACH_committment**	3.40	*.85*	3.54	*.79*	3.43	*.94*
**TEAM_learning**	3.51	*.68*	3.49	*.78*	3.47	*.76*
**TEAM_effort**	3.87	*.60*	3.79	*.68*	3.72	*.71*
**TEAM_intra-compet**	3.15	*.62*	3.33	*.67*	3.41	*.75*
**TEAM_intra-conflict**	2.63	*.70*	2.79	*.96*	3.05	*.85*

Table 18 - One Way ANOVA results

PWB composite	*df*	*SS*	*MS*	*F*	*p.*
Between groups	2	17.852	8.926	11.079	.001
Within groups	378	318.266	.842		
Total	380	336.118			

Table 19 - One Way ANOVA results for PWB composite

PWB Autonomy	*df*	*SS*	*MS*	*F*	*p.*
Between groups	2	44.205	22.102	9.714	.001
Within groups	394	875.805	2.223		
Total	396	920.010			

Table 20 - One Way ANOVA results for Autonomy

PWB Meaning	*Df*	*SS*	*MS*	*F*	*p.*
Between groups	2	61.421	30.711	13.321	.001
Within groups	401	1105.161	2.756		
Total	403	1166.582			

Table 21 -One Way ANOVA results for Meaning

Parent Worry	*df*	*SS*	*MS*	*F*	*p.*
Between groups	2	5.259	2.629	6.463	.002
Within groups	367	149.308	.407		
Total	369	154.566			

Table 22 - One Way ANOVA results for Parental Worry

Parent Learning	*df*	*SS*	*MS*	*F*	*p.*
Between groups	2	3.469	1.735	6.174	.002
Within groups	367	103.120	.281		
Total	369	106.590			

Table 23 - One Way ANOVA results for Parental Learning

Coach Closeness	*df*	*SS*	*MS*	*F*	*p.*
Between groups	2	7.228	3.614	6.041	.003
Within groups	408	244.090	.598		
Total	410	251.318			

Table 24 - One Way ANOVA results for Coach Closeness

Intra team conflict	*df*	*SS*	*MS*	*F*	*p.*
Between groups	2	11.122	5.561	7.601	.001
Within groups	399	291.915	.732		
Total	401	303.036			

Table 25 - One Way ANOVA results for Intra Team Conflict
